# Intraoperative Radiotherapy for Breast Cancer

**DOI:** 10.3389/fonc.2017.00317

**Published:** 2017-12-22

**Authors:** Eleanor E. R. Harris, William Small

**Affiliations:** ^1^Department of Radiation Oncology, Brody School of Medicine, East Carolina University, Greenville, NC, United States; ^2^Department of Radiation Oncology, Stritch School of Medicine, Loyola University, Chicago, IL, United States

**Keywords:** breast cancer, intraoperative radiotherapy, breast conservation therapy, partial breast irradiation, radiation therapy

## Abstract

Intraoperative radiotherapy (IORT) for early stage breast cancer is a technique for partial breast irradiation. There are several technologies in clinical use to perform breast IORT. Regardless of technique, IORT generally refers to the delivery of a single dose of radiation to the periphery of the tumor bed in the immediate intraoperative time frame, although some protocols have performed IORT as a second procedure. There are two large prospective randomized trials establishing the safety and efficacy of breast IORT in early stage breast cancer patients with sufficient follow-up time on thousands of women. The advantages of IORT for partial breast irradiation include: direct visualization of the target tissue ensuring treatment of the high-risk tissue and eliminating the risk of marginal miss; the use of a single dose coordinated with the necessary surgical excision thereby reducing omission of radiation and the selection of mastectomy for women without access to a radiotherapy facility or unable to undergo several weeks of daily radiation; favorable toxicity profiles; patient convenience and cost savings; radiobiological and tumor microenvironment conditions which lead to enhanced tumor control. The main disadvantage of IORT is the lack of final pathologic information on the tumor size, histology, margins, and nodal status. When unexpected findings on final pathology such as positive margins or positive sentinel nodes predict a higher risk of local or regional recurrence, additional whole breast radiation may be indicated, thereby reducing some of the convenience and low-toxicity advantages of sole IORT. However, IORT as a tumor bed boost has also been studied and appears to be safe with acceptable toxicity. IORT has potential efficacy advantages related to overall survival related to reduced cardiopulmonary radiation doses. It may also be very useful in specific situations, such as prior to oncoplastic reconstruction to improve accuracy of adjuvant radiation delivery, or when used as a boost in higher risk patients to improve tumor control. Ongoing international clinical trials are studying these uses and follow-up data are accumulating on completed studies.

## Introduction

Partial breast irradiation has been established as a suitable treatment option for appropriately selected women with early stage breast cancer by numerous clinical trials dating back to the 1990s. There are several techniques which have been studied to accomplish irradiation of the periphery of the lumpectomy bed as sole therapy after lumpectomy, which is the target volume for any form of partial breast treatment. Intraoperative radiotherapy (IORT) is one such technique. The major difference between IORT techniques and other forms of APBI is timing of the procedure. IORT is most often performed at the time of breast surgery as a single dose, while other APBI techniques are performed post-operatively, using target volumes are typically based on CT images and delivering multiple fractions. IORT requires specialized radiotherapy equipment, and there are several technologies available to provide IORT partial breast irradiation, which deliver treatment with either electrons or 50 kV X-rays. IORT has the advantage of completing the breast-conserving surgery and, in most cases, the partial breast irradiation as one combined procedure. All forms of APBI treat a smaller volume of normal tissue than whole breast radiation (WBRT), thereby reducing the potential lung and cardiac toxicities of radiation treatment, and reducing the overall treatment time compared with whole breast irradiation. IORT has the additional advantage of delivering a single dose at the time of surgery, potentially reduces non-compliance to post-operative radiation, and mastectomy rates among women without ready access to a radiotherapy center. There are two recently published large prospective randomized controlled trials comparing post-lumpectomy standard whole breast irradiation to IORT, one using electrons and one using 50 kV photons, which have shown low-local recurrence rates for IORT with acceptable toxicity and excellent overall survival outcomes. These trials begin to inform our knowledge of selection criteria for optimal breast IORT candidates and provide the first evidence of outcomes and toxicity when using these techniques. Patient selection is important when recommending IORT, as the final pathology is not available at the time of treatment, so in order to avoid the potential use of subsequent whole breast irradiation, careful pre-operative, and intraoperative assessment can help ensure that high-risk features such as positive margins or positive sentinel nodes are minimized. As all techniques of partial breast irradiation leave some volume of the breast unirradiated, understanding of the selection criteria for each of the various techniques is critical information for clinicians when considering which patients may be appropriately treated with IORT or any other APBI technique. This review will discuss the clinical trial data, patient selection criteria, advantages and disadvantages of partial breast IORT, and published guidelines.

## Radiobiology and Tumor Microenvironment

A common calculation for assessing the efficacy of different dose fractionations in radiotherapy is the relative biological effectiveness (RBE = *D_x_*/*D*). This parameter allows comparisons of radiation-induced cellular damage at a designated dose (*D*) relative to a reference dose (*D_x_*) for the selected endpoint, such as percentage of cells surviving after 2 Gy (SF2). It is well known that the RBE of photons increases with decreasing energy, explained by a decrease in energy of secondary electrons with an increase in linear energy transfer (1, 2). Cell culture experiments utilizing cell survival methods such as SF2 confirm enhanced biological effects after exposure to lower energy X-rays. Brenner et al. modeled RBE at clinically relevant doses, at which the RBE for 40 kV photons was about 1.4 compared with 4 mV. Since effective RBE increases with depth this creates a less rapid fall-off of the biologically weighted dose at measured depth. A published review summarized RBEs for 10% cell survival established using different systems and tumor cell types for low energy X-rays (10–240 kV) to range from 1.1 and 1.7 (3). The RBE of 50 kV electronic brachytherapy sources has been estimated to exceed biological effectiveness by 40–50% over Co^60^ or Ir^192^ (4). Other investigators have employed a linear-quadratic formula to model the RBE of 50 kV X-rays modeled as an equivalent to a fractionated dose of 2 Gy (EQD2) as a function of depth, with the probability of local control estimated from clinical dose response data (5). This model resulted in a theoretical “sphere of equivalence” to explain the improved tumor control probability in the high-dose cloud near the applicator surface, where residual microscopic disease is most likely to be located, and compensating for a somewhat lower tumor control probability as the distance from the applicator increases. Overall local control patterns thus exhibited a different spatial pattern but were ultimately very similar to conventionally fractionated external beam irradiation. In the example of IORT for breast cancer, higher RBE for low-energy X-rays may result in higher tumor control rates in the breast tissue in closest proximity to the surgical excision bed and effectively eliminating the “marginal miss.” In addition, cell culture data suggest that the RBE decreased at increasing distance, potentially reducing the effective dose to adjacent critical structures including heart and lung (6). The tumor bed intraoperatively is better oxygenated, which may also improve cell kill probability. There may also be some radiobiological advantages in using a higher dose per fraction in breast cancer, which has been estimated to have an alpha/beta ratio of around 4, therefore may demonstrate a higher radioresponsiveness to higher doses per fraction (7). The biologically equivalent dose (BED) for an alpha/beta of 4 in the linear-quadratic model for a prescribed single dose of 10 Gy is isoeffective to about 24 in 2 Gy fractions.

The microenvironment of the breast cancer cells likely plays a critical role as well in the risk of tumor recurrence, and this microenvironment is altered by the use of immediate radiation peri-operatively. Belletti et al. collected wound fluid from the lumpectomy cavity over 24 h after surgery, half of whom had IORT at the time of lumpectomy (8). The wound fluid was used to stimulate several breast cancer and control cell lines and analyzed for cell growth and motility. Normal wound fluid stimulated proliferation, migration, and invasion in breast cancer cell lines, while these effects were abrogated by wound fluid from immediately irradiated samples. The radiated wound fluid had altered expression cytokines, suggesting that the radiation had altered protein expression. Fabris et al. collected tissues from irradiated using IORT and non-irradiated tissue from breast cancer patients after surgery, and profiled the tissue for microRNA expression (9). IORT radiation changed the wound response by inducing expression of miRNA 223 in the peri-tumoral tissue, which downregulated expression of epidermal growth factor (EGF) and EGF receptor activation. This downregulation cascade prevented breast cancer cell growth and reduced local recurrence in mice models. A number of other studies have noted the stimulatory effect of wound fluid on breast cancer cells, suggesting a role of the fluid in cancer cell proliferation and possibly local recurrence, an effect that may be muted by immediate radiation, with its abrogating effect on protein expression. Clinical trial data are clinically consistent with these concepts and investigations of the biological effects of immediate high-dose radiation on the wound fluid and immunologic environment are ongoing.

## IO(E)RT Technologies

The Intrabeam^®^ system (Carl Zeiss Meditec, Dublin, CA, USA) uses a 50 kV photon beam mobile X-ray unit that has been in clinical use since 1999 (10). The miniaturized accelerator produces an electron beam that is accelerated to the tip of a drift tube generating an isotropic point source of low-energy X-rays. The source is permanently integrated into the treatment unit, and is calibrated daily and externally yearly. This system has been designed for single fraction IORT and is calibrated at a single dose rate and output factor. IORT is delivered using multiuse solid state spherical applicators of size ranges 1.5–5 cm diameter. Treatment time typically runs 20–45 min depending upon the applicator size used.

The Axxent^®^ System (Xoft Inc., Sunnyvale, CA, USA) is an electronic brachytherapy machine in clinical use since 2009 (11). The radiation source is a miniature, electronic, high-dose rate low-energy X-ray tube integrated into a flexible multi-lumen catheter producing 40–50 Kv X-rays at the catheter tip. The Axxent system was originally designed for fractionated balloon-based partial breast irradiation using variable currents and voltages to allow for changes in dose rates or depths, and can also be used for single fractions. The source is disposable and used for up to 10 fractions, with disposable balloons of spherical and ellipsoidal sizes (3–6 cm spherical and 5–6 × 7 cm elliptical), over treatment times of 10–20 min. Both technologies can be used in a standard operating room with portable shielding only.

Mobile electron accelerators use electrons energies ranging from 3 to 12 MeV. The lumpectomy cavity of the breast is treated with a cone inserted intraoperatively (12). Electrons are more penetrating than low-energy X-rays, requiring breast tissue to be mobilized and for shields to be inserted into the posterior lumpectomy cavity in order to shield tissues inside the thorax. Doses of 20–21 Gy usually delivered at a low-electron energy for the measured depth. This technology is often notated as intraoperative electron radiation therapy (IOERT).

## IO(E)RT as Sole Partial Breast Radiation

To date, there are two large prospective randomized trials published using breast IO(E)RT, the TARGIT-A trial and the ELIOT trial. TARGIT-A compared conventional WBRT (EBRT) to single dose IORT (TARGIT) and enrolled 3,451 patients from 33 centers in 10 countries between the years 2000 and 2012. This study used a non-inferiority statistical design which anticipated a 15% probability of adverse pathologic features on final pathology leading to additional WBRT after initial IORT (13). There were pre-specified strata described as IORT at the time of lumpectomy (pre-pathology), or IORT performed during a subsequent procedure at a different time (post-pathology).

Women enrolled to TARGIT-A were age 45 years or older with operable unifocal invasive ductal carcinoma. Per eligibility criteria pathologic findings requiring subsequent WBRT after IORT included positive excision margins, extensive intraductal component, or the presence of invasive lobular carcinoma. Participating centers could prospectively specify additional other factors. Overall, 22% of women enrolled in the pre-pathology and 3.6% of those in the post-pathology strata received additional WBRT after randomization to IORT. The majority enrolled had lower risk pathologic features, including ≤2 cm (87%), low to intermediate grade (85%), negative nodes (84%), estrogen receptor positive (93%), and mammographic detection (63%). With median follow-up of 2.5 years of the whole cohort and over 1,200 patients with 5-year median follow-up, for the primary endpoint of in-breast recurrence (IBR), the investigators reported 5-year IBR in the EBRT arm of 1.3%, and in the TARGIT arm of 3.3%, a 2% difference which was within the pre-specified 2.5% non-inferiority margin (*p* = 0.042). The impact of the timing of IORT was analyzed between the two strata. For pre-pathology stratum, IBR was 1% in the EBRT arm and 2.1% in the TARGIT arm (*p* = 0.31). For post-pathology stratum, IBR was 1.7% in the EBRT arm and 5.4% in the TARGIT arm (*p* = 0.069). These findings prompted the trialists to conclude that pre-pathology timing was more optimal. Overall survival was higher in the TARGIT arm (3.9%) compared with the EBRT arm (5.3%; *p* = 0.099), mainly due to higher rates of cardiopulmonary deaths. Toxicity comparisons including hematoma requiring treatment, post-operative infection, delayed wound healing, and all major toxicities were similar between the two arms.

The largest series outside of the TARGIT-A trial using 50 kV IORT has been reported by the TARGIT-R North American multi-institutional IORT retrospective registry trial (14). Nineteen institutions participated in this registry and reported outcomes on 822 women treated from 2007 through 2013 with minimum 6 months follow-up and a median follow-up of 2 years. Registrants were treated with IORT without (*n* = 537) or with WBRT (*n* = 110) or IORT as intended boost (*n* = 115). As is typical of APBI studies and registries, patients were mainly lower risk, or meeting American Society for Radiation Oncology (ASTRO) 2009 “suitable” criteria, with a median age of 67, <2 cm (90%), estrogen receptor positive (91%), Her2 non-amplified (89%), grade 1–2 (83%), without lymphovascular invasion (91%), and sentinel node negative (89%). Interestingly, 52% of registrants had a pre-operative breast MRI performed. Post-operative WBRT was recommended in 17% of IORT patients due to unfavorable pathologic findings, and 14% of registrants received IORT as a planned boost. A small number (*n* = 60) had delayed IORT as a second procedure rather than at the time of lumpectomy. Local IBRs were seen in 2.3% (*n* = 19 of 822), and axillary nodal recurrences in 0.2% (*n* = 2), at a median time to recurrence of 19 months. One death was attributed to breast cancer. Local recurrence by type of IORT was reported as follows: IORT alone, 2.4%; secondary IORT, 6.6%; IORT + WBRT, 1.7%; IORT boost, 1.8%. Thirteen of 19 local recurrences occurred >1 cm from the lumpectomy site. Several of these patients had higher risk features including ER negative tumors or positive sentinel nodes but elected not to undergo WBRT. Complications were low, including post-operative seroma in 9%, hematoma in 1.5%, and infection requiring antibiotics in 2.8%. The early results of this large retrospective registry are similar to those seen in the TARGIT-A randomized trial. There is also an ongoing prospective United States registry study, TARGIT-US (15), which should complete accrual this year.

The ELIOT study had a similar design to the TARGIT-A, but used mobile electron technology to deliver IORT. It was a single institution study completed by the Institute Milan. This trial randomized 1,305 women between the years 2000 and 2007 between external conventional whole breast irradiation (EBRT) (50 + 10 Gy boost) and single dose electron IOERT (21 Gy) with no additional WBRT (16). The study statistical design was an equivalence endpoint, with a pre-specified margin for local recurrence of 7.5% after IOERT. Women eligible for the study had stage I–II invasive breast cancer up to 2.5 cm in size between ages 48 and 75 years old. Lower risk tumors were pre-dominant, with patient characteristics including estrogen receptor positive in 90%, Her2 negative in 97%, and negative nodes in 74%. With median follow-up of 5.8 years, 5 years IBR was recorded in an unexpectedly low percentage in the EBRT arm (0.4%) as well as in the IOERT arm (4.4%), which was within the pre-specified equivalence margin of 7.5% (*p* < 0.0001). There was no difference in overall survival (96.8 and 96.9%, respectively; *p* = 0.59). Cutaneous toxicities were significantly better for all recorded endpoints in the IOERT arm, although a higher incidence of fat necrosis was seen after IOERT, with no overall differences in other side effects including breast fibrosis, retraction, pain, or burning. The ELIOT trialists concluded that the unselected population helped to define stricter selection criteria which could result in a lower IBR rate. They identified risk factors associated with local recurrence after IOERT as tumor size >2 cm, grade 3, 4, or more positive nodes, and triple negative histology.

The published randomized trials and large multi-institutional studies, provide guidance in selecting appropriate patients for breast IORT, and provide the basis for guidelines and consensus statements of selection criteria. The ASTRO consensus guideline was updated in 2017 to include a key question on breast IO(E)RT. The statement notes that IO(E)RT use should be restricted to women who otherwise meet “suitable” criteria for partial breast irradiation, and Coverage with Evidence Development on a registry or trial applies to low-energy X-ray IORT while awaiting longer follow-up on accrued clinical trials (17). The limited follow-up time was a major concern of this panel. There have been published commentary which disagree with some of the conclusions of the ASTRO consensus statement on IORT, including by a group representing other professional societies and IORT users, which highlighted inconsistencies in interpretation of the TARGIT-A data (18). TARGIT investigators advocate use of the study criteria and offering patients WBRT as was done on the study when pathologic features indicative of more diffuse disease are present on final pathology. Similarly, the ELIOT investigators discussed the potential for use of stricter selection criteria for IOERT than used in the trial, with initial use or inclusion of WBRT when those features are present on final pathology. With any type of IO(E)RT, radiation most commonly delivered at the time of surgery when final pathologic features are not yet available, therefore selection criteria should be based on the information available prior to as well as during surgery. Many surgeons and radiation oncologists prefer to have rigorous intraoperative assessment of sentinel nodes and margins to assist in decision making and patient selection.

### Toxicity and Cosmesis

In studies using 50 kV X-rays, several have reported acute and late toxicity profiles after IORT ± WBRT. In the original TARGIT-A publication, all clinically significant complications occurred in 3.3% or fewer patients, including hematoma or seroma requiring intervention, infection, wound healing, or any grade 3 toxicity, and were similar between IORT and WBRT arms. In the study update published in 2013, it was noted that complications at 6 months showed no difference between arms for any wound-related complications, with fewer grade 3–4 skin toxicities after IORT. Keshtgar reported cosmesis up to 4 years after IORT on a TARGIT-A subprotocol as assessed by photograph-analyzing software. IORT patients were about twice as likely to have excellent or good cosmetic scores as WBRT treated patients (19). One German institution examined their 48 TARGIT-A enrolled patients in a subgroup analysis of post-treatment mammogram findings (20). They noted a higher rate of radiographic fat necrosis after IORT (56%) than after conventional WBRT (24%), and more scar calcifications as well. Sperk et al. noted no differences between IORT ± WBRT versus WBRT with respect to fibrosis, breast edema, lymphedema, pain, or hyperpigmentation (21). Fibrosis was higher after IORT + WBRT (37.5%) than after IORT alone (6%) or WBRT only (18%) at 3 years. Telangiectasias were not seen in any IORT only patients, compared with 17% of women after WBRT ± IORT. The Copenhagen group conducted a subgroup analysis of post-treatment pain in their enrolled TARGIT-A cohort (*n* = 244) conducted using patient reported outcomes data, and found that persistent pain was reported in 34% of WBRT patients compared with 25% of IORT patients (22).

In studies using electron IOERT, the ELIOT trial reported on the subset of patients in whom toxicity data were available, noting that side effect profiles significantly favored IOERT compared with WBRT, especially as related to skin toxicity, with less erythema, hyperpigmentation, dryness, and pruritis (all *p* < 0.04). No differences were recorded for fibrosis, pain, or burning sensation. Only radiologic presence of necrosis was higher in the IOERT group. The ELIOT group randomly selected 119 patients treated with sole IOERT for further late toxicity assessment using standard rating scales (23). At a median of 6 years, grade 2 fibrosis was noted in 32% and grade 3 fibrosis in 6%. Excellent or good cosmesis was scored by patients in 77% and by physicians in 84%. The Netherlands group compared institutional data for women treated with IOERT (*n* = 26) or conventional WBRT (*n* = 45) based on seven asymmetry features (24). Features favoring IOERT with smaller differences between treated and untreated breast included breast contour, relative breast area, and breast overlap. Excellent and good cosmesis after IOERT was scored as 88% by patients and 96% by physicians.

### Quality of Life

Increasingly, study of quality of life accompanies more traditional outcomes endpoints and influences patient preferences and informed consent discussions for patients and their providers facing breast cancer treatment. Investigators in Australia conducted a survey of Western Australia breast cancer health professionals and reported that among those surveyed, 3–7.5% considered breast IORT unacceptable treatment at any risk of local recurrence, 18–21% considered IORT acceptable at risks equivalent to that of WBRT, while 56–59% considered IORT acceptable if associated with a 1–3% increased local recurrence risk (25). In a survey of patient preference considering treatment options of breast IORT or fractionated multi-week whole breast irradiation which described to participants alternative increases in rates of local recurrence risk over 10 years, this survey found that patients accepted a median increase in local recurrence risk for IORT of 2.3% (26). In addition, 91% surveyed would accept IORT if the treatment were equivalent or associated with a slightly higher risk of local recurrence compared with WBRT. Another quality of life study among a subgroup of German patients enrolled in the TARGIT-A trial administered the EORTC QLQ-C30 and BR23 survey instruments (27). Patients in the IORT alone arm indicated significantly less general pain, breast, and arm symptoms and better overall functioning than patients in the WBRT arm.

## Breast IO(E)RT in Other Clinical Contexts

Intraoperative radiotherapy, as with all types of APBI, is less well studied in conjunction with breast-conserving surgery for ductal carcinoma *in situ* (DCIS). The recent update of the ASTRO APBI consensus statement now classifies APBI for pure DCIS as “suitable” when meeting certain specific criteria. A California group has published the only series to date using 50 kV IORT for patients with DCIS. In this series, selection criteria included tumor size <4 cm on pre-operative imaging with pure DCIS on biopsy and deemed resectable with breast conservation (28). Thirty-five patients had IORT, with a mean tumor size of 1.5 cm. Five patients had close or positive margins, two of whom had mastectomy due to extent of DCIS in the specimen, and three had re-excision followed by WBRT, for a 14% rate of additional treatment after surgery plus IORT. The 3-year local recurrence rate was 5.7%.

A study from the Milan group used IOERT in conjunction with nipple-sparing mastectomy, comparing 800 patients receiving IOERT to the retroareolar region of the nipple to 201 patients with nipple-sparing mastectomy followed by delayed one-dose radiation later (29). At median follow-up of 20 months, they noted nipple-areolar necrosis in 3.5%, and nipple removal in 5%. Of the 1.4% local recurrences, none were seen in the nipple, but at the site of the primary tumor. No difference in outcomes was noted between the two techniques. This report does not discuss any comparison with patients who have not received any radiation to the nipple-areolar complex. A second series using 50 kV IORT single dose of 16 Gy after nipple-sparing mastectomy describes only seven patients with 7 months follow-up, with no acute toxicity attributable to radiation, no necrosis of the nipple complex or local recurrences (30).

In these special clinical scenarios, further data are needed to define the appropriate role of IORT. The direct visualization and elimination of the possibility of marginal miss makes IORT an attractive technique in situations where accuracy of dose targeting is particularly critical, such as women with higher risk cancers, as being investigated in the TARGIT-B trial of IORT versus conventional boost. Areas for further study of the efficacy and toxicity of IORT may include as sole therapy for lower risk DCIS, as a boost prior to planned oncoplastic reconstruction, and as part of re-treatment after prior whole breast irradiation for limited local recurrence.

## IO(E)RT Boost

A boost dose to the periphery of the surgical lumpectomy bed has been shown to further lower the risk of local recurrence, especially for younger women, women with higher grade, triple negative or larger tumors, and those with positive margins or extensive lymphovascular invasion (31). The tissue in closest proximity to the primary cancer has the highest density of residual microscopic cells and is therefore at highest risk for local recurrence. Several institutional studies have reported using IORT as a boost with planned WBRT to follow. The theoretical advantages of using IORT as a boost include the ability to directly visualize the tumor bed and thereby avoid marginal misses when boosting a CT-based volume. The same BED, oxygenation, and biological advantages theoretically present for single dose IORT may be relevant for IORT boost as well, and are under investigation. Several studies using either IOERT (intraoperative electrons) or 50 kV IORT as a boost have been reported. Ongoing studies going IORT boost include the TARGIT-B(oost) (32) and HIOP trials (33).

Intraoperative electron radiation therapy boost has been reported as a pooled analysis by the International Society of Intraoperative Radiotherapy (34). In this analysis, 1,109 unselected patients from seven European centers, 60% of whom had at least one high risk factor, were treated similarly with IOERT boost at a median dose of 10 Gy and a subsequent whole breast dose of 50–54 Gy. After a median follow-up of 5 years, the local recurrence risk was 0.8%, half seen in the index quadrant. Risk factors for recurrence included high grade, age under 40, and ER negative. Upon examining the impact of delays from IOERT boost to WBRT, no impact on local recurrence of delays up to 140 days was seen. The Salzburg IOERT group conducted a matched-pair analysis of IOERT boost and external electron boost patients, who had IBR rates at 10 years of 1.6 and 7.2%, respectively (35).

Low-energy X-rays IORT as a boost has been reported in two cohort series. One multicenter pilot study treated with 20 Gy to cavity surface intraoperatively followed by 45–50 Gy whole breast in 299 women undergoing lumpectomy. After a median follow-up of 5 years, the observed local recurrence rate was 2.7% (36). A single institution series of 197 patients received an IORT boost of 18–20 Gy then 46–50 Gy whole breast, reporting a 5-year local relapse free survival of 97% (37).

Regardless of technique the toxicity of IORT boost appears to be acceptable. The virtual complete skin sparing associated with use of IORT is likely to have a positive impact on any cutaneous toxicity profiles. Acutely there are no reports of increased post-operative infections or delayed wound healing. Late fibrosis has been reported to range from about 0 to 15% for grade 3 toxicity, depending upon technique and dose. Cosmesis does not seem to be compromised in the studies described when compared with conventional boost techniques, although assessment tools have varied. Lemanski has reported the longest term experience in using IOERT boost, reporting 9-year outcomes on 50 women receiving 10 Gy IORT then 50 Gy whole breast, with no grade 3 fibrosis and 14% grade 2 fibrosis (38). Mayo Arizona conducted a prospective study of 10 Gy IOERT then 48 Gy to the whole breast, and reported a 3.8% 6-year local recurrence rate, with excellent or good cosmesis in 87% of patients (39). An Australian group reported that 55 patients treated with 5 Gy at 1 cm IORT boost then 50 Gy whole breast had no local recurrences at 3 years, but grade 3 fibrosis was 15% (40). There is one report of IOERT boost (12 Gy) followed by hypofractionated whole breast (37.05 Gy in 13 daily fractions of 2.85 Gy) in 204 pre-menopausal women. Reporting only acute toxicity, grade 3 skin toxicity was 4%, grade 2 skin toxicity was 29%; late skin toxicity at 12 months was grade 3 and 4 in one patient each (41).

For context in comparison, the EORTC boost trial, the 5- and 10-year rates of moderate to severe fibrosis was reported in 11 and 28% of boost patients compared with 10 and 13% on the no boost arm. In this study, at 3 years, excellent and good cosmesis was somewhat worse in the boost arm compared with no boost, 71 versus 86%, respectively (42, 43).

There are several reports of using IORT boost in conjunction with breast-conserving surgery and WBRT in special patient cohorts. One retrospective series reported on IORT boost after neoadjuvant chemotherapy in 61 patients and a contemporaneous consecutive cohort of 55 patients who received a conventional external beam boost, all receiving WBRT (44). At 4 years follow-up, there was no difference in 5-year local recurrence (9.8% with IORT and 8.3% with external boost), and there was a better overall survival in the IORT arm (97 versus 82%) related to fewer non-breast cancer deaths. The Salzburg group has also reported IORT boost in a retrospective series of triple negative patients undergoing breast conservation, with an 8-year actuarial local recurrence rate of 11%, all of those recurrences occurring in high-grade cancers (45).

A German group has reported on IORT boost in the setting of oncoplastic reconstruction. This is a particularly appealing clinical scenario for the use of an IORT boost, as the oncoplastic reconstruction which immediately follows the definitive oncologic surgery to remove the cancerous cells can eradicate any clear delineation of the tumor bed and preclude the use of external beam boost, causing some patients to be underdosed. Performing IORT after the lumpectomy but prior to the oncoplastic rearrangement eliminates the risk of target volume miss, the possibility of dissemination of microscopic disease during the reconstruction, or inability to identify the boost target on post-operative treatment planning image sets. However, minimal data exist to support the efficacy of this approach. The Cologne group has used IORT boost in 149 patients who also underwent an oncoplastic reconstruction (glandular rotation or mammoplasty), and have reported only post-operative toxicity, with a 2% seroma formation rate. Additional outcome and efficacy data from this and other series will be welcome (46).

## Conclusion

Breast IO(E)RT is currently primarily a technique for partial breast irradiation which has been well established as an option for patients who are otherwise appropriate candidates for APBI. Two large randomized trials, the TARGIT A trial establishing 50 kV IORT and ELIOT establishing electron based IOERT, both have published excellent results regarding local control and acceptable toxicity. While local recurrence was slightly higher after IORT in both studies, it was within the clinically relevant range seen in numerous other clinical trials of various radiation techniques, including APBI and WBRT approaches (see Table 1). Patient preference analyses have shown that some women will accept the risk of a small increase in local recurrence in order to preserve their breast, to mitigate toxicity or to reduce the burden of lengthier radiation treatment courses. Overall survival in TARGIT-A was significantly better in the IORT group, an intriguing observation that warrants additional investigation. It is appropriate for these considerations to be discussed with patients as part of the clinical decision making and informed consent process for radiation treatment.

**Table 1:** Prospective randomized controlled trials of partial breast irradiation compared to whole breast radiation.

Study	Author	PBI technique	Number patients	Med F/U (years)	Age > 50	pT1 %	pTis %	pN0 %	ER+ %	5-Year local recurrence	5-Year overall survival
Hungary	Polgar et al. (47, 48)	MC Brachy (69%) or electrons (31%)	128	10.2	77%	100	0	94.5	92	5 years: 4.7%, 10 years: 5.9%	5 years: 94.6%, 10 years: 80%
		WBRT	130		75%	100	0	94.6	88	5 years: 3.4%, 10 years: 5.1%	5 years: 91.8%, 10 years: 82%
Florence	Livi et al. (49)	IMRT	260	5.0	84%	86	9	89	95	1.5%	99.4%
		WBRT	260		83%	82	12	82	96	1.5%	96.6%
GEC-ESTRO	Strnad et al. (50)	MC Brachy	633	6.6	86%	84	6	94	92	1.44%	97.3%
		WBRT	551		83%	86	4	95	91	0.92%	95.6%
TARGIT-A	Vaidya et al. (13)	IORT ± WBRT	1,721	2.5	>45, 98%	96	0	82	90	3.3%	96.1%
		WBRT	1,730		99%	95	0	84	93	1.3%	94.7%
ELIOT	Veronesi et al. (16)	IOERT	651	5.8	93%	87	0	74	90	4.4%	96.8%
		WBRT	654		93%	84	0	73	91	0.4%	96.9%

*PBI, partial breast irradiation; MC, multicatheter; Brachy, brachytherapy; WBRT, whole breast radiation; IORT, intraoperative radiotherapy; IOERT, intraoperative electron radiotherapy; IMRT, intensity modulated radiotherapy; F/U, follow-up; ER, estrogen receptor; pTis, pathologic ductal carcinoma in situ; NR, not reported*.

When considering use of IO(E)RT techniques for partial breast treatment after lumpectomy, it is recommended to select patients who fall into the low-risk categories among published guidelines, using the “suitable” or “good risk” criteria for patients who are general candidates for APBI. IO(E)RT has potential advantages over external or brachytherapy-based techniques given the direct visualization of and contact with the target tissue and the immediacy of treatment, but has the disadvantage of lacking final pathologic assessment of the margins and sentinel nodes, placing a percentage of women at risk of being recommended to undergo additional external beam irradiation. Therefore, when intended to be used for partial breast treatment, patient selection should focus on clinicopathologic factors predictive of negative nodes and negative margins. Careful assessment of pre-operative mammographic and other imaging studies for features, such as extent of calcifications, may be helpful. Intraoperative techniques can be useful as well, including assessment of margins and sentinel nodes intraoperatively, and careful excision technique to maximize clear margins, such as taking additional shave margins as needed.

Potential emerging indications for breast IO(E)RT under investigation include as a boost in higher risk patients, as a boost in patients undergoing oncoplastic reconstruction, as sole therapy for lower risk DCIS, and as re-treatment for IBR after prior breast irradiation. Longer term follow-up on the completed trials is anticipated and ongoing studies and registries will help define these new modalities.

## Author Contributions

EH wrote manuscript, created table, completed literature review. WS wrote sections of manuscript and edited entire manuscript.

## Conflict of Interest Statement

EH and WS are both members of the TARGIT-US Scientific Steering Committee, which is not associated with any financial remuneration. EH received travel funds from the TARGIT Collaborative Group to give an invited lecture in 2017. WS has received travel reimbursement from and is on a speaker’s bureau for Carl Zeiss Meditec.
